# The Key Network of mRNAs and miRNAs Regulated by HIF1A in Hypoxic Hepatocellular Carcinoma Cells

**DOI:** 10.3389/fgene.2022.857507

**Published:** 2022-06-14

**Authors:** Tong Liu, Jing Tang, Xiaoyu Li, Yuan Lin, Yuma Yang, Kai Ma, Zhaoyuan Hui, Hong Ma, Yanyan Qin, Hetian Lei, Yanhui Yang

**Affiliations:** ^1^ Ningxia Key Laboratory of Prevention and Control of Common Infectious Diseases, The School of Basic Medical Sciences, Ningxia Medical University, Yinchuan, China; ^2^ Shenzhen Eye Hospital, Shenzhen Eye Institute, Jinan University, Shenzhen, China

**Keywords:** HIF1A, CRISPR/Cas9, HepG2, mRNA-seq, miRNA-seq

## Abstract

**Purpose:** Hypoxia plays an essential role in the progression of hepatocellular carcinoma (HCC), whereas hypoxia inducible factor-1 (HIF-1) is the key transcription factor allowing HCC to survive hypoxia. The aim of this study was to define the essential mRNAs and miRNAs regulated by HIF1A and dissect their functions, interactions, and tumor-infiltrating immune cells in HCC.

**Methods:** A human HCC cell line HepG2 was used as a cell model of HCC. The CRISPR/Cas9 system was used to knock out *HIF1A* in HepG2 cells, and RNA sequencing was utilized to characterize differentially expressed mRNAs and miRNAs in the *HIF1A*-knockout HepG2 cells; the identified candidates were then analyzed by GO annotation and KEGG pathway enrichment to study their function and establish a PPI network. Quantitative (q) PCR was used to verify if there were significant differences in the expression of mRNAs, and the association of the selected mRNAs expression with immune cell infiltration levels was further analyzed using The Cancer Genome Atlas (TCGA) pan-cancer data.

**Results:** Using RNA-sequencing, we discovered that there were 1535 mRNAs differentially expressed (adjusted *p* < 0.05, |fold change|>1.5) in the *HIF1A*-knockout HepG2 cells, among which there were 644 mRNAs upregulated and 891 mRNAs downregulated. GO annotation and KEGG pathway enrichment showed that these mRNAs were involved in glycolysis/gluconeogenesis, PI3K-Akt signaling pathways, and HIF-1 signaling pathways. In addition, we found that there were 309 miRNAs differentially expressed (adjusted *p* < 0.05, |fold change|>1.5) in the HIF1A-knockout HepG2 cells, of which there were 213 miRNAs upregulated and 96 miRNAs downregulated. Our further analyses uncovered that these miRNA putative targets were involved in the hippo signaling pathway, axon guidance, and tight junction. Moreover, the construction and analysis of the PPI network showed that *OASL*, *IL6*, and *TAF1* were recognized as hub genes with the highest connectivity degrees. Importantly, in the *HIF1A*-knockout HepG2 cells, our qRT-PCR data confirmed the selected mRNA changes revealed by RNA-sequencing, and with TCGA pan-cancer data, we revealed that the expressional levels of these three genes, *LUM*, *SCOC*, and *CCL2*, were associated with immune cell infiltration levels.

**Conclusion:** The identified potential key network of mRNAs and miRNAs regulated by *HIF1A* in the HCC cells suggests a key role of *HIF1A* in the tumorigenesis of HCC.

## Introduction

Hepatocellular carcinoma (HCC) is a common cancer worldwide, with a global incidence of more than 600,000 new cases per year (Bray et al., 2018), and ranks the fifth in incidence and third in mortality worldwide. In addition, more than 60% of patients are diagnosed at advanced stages with a 5-year survival rate of less than 10% (Cai and Liu, 2021).

The initiation and progression of HCC is not fully understood so far, while hypoxia is known to contribute to the development of HCC (Parks et al., 2016). The hypoxic microenvironment regulates tumor angiogenesis and energy metabolism (Gao et al., 2007), and it is linked to treatment resistance of cancer and poor prognosis of patients (Tsai and Wu, 2012; Ramapriyan et al., 2019).

HIF1A is a transcription factor that is required for a tumor to adapt to hypoxia (Cheng et al., 2007; Semenza, 2012a). It can decrease the maturation of dendritic cells (DCs) by promoting the production of vascular endothelial growth factor (VEGF) (Lin et al., 2004; Zhang et al., 2005), and it can also regulate tumor cell energy metabolism programs and regulate cell cycle checkpoint proteins (Semenza, 2012b; Hu et al., 2019). Tumors can adapt to the hypoxic environment, while the relevant immune cells cannot survive in the hypoxic environment, making the tumor escape the immune system and promoting the growth of tumor tissues (Noman et al., 2014; Xu et al., 2016).

The CRISPR system is a special family of DNA repeats that are widely distributed in bacterial and archaeal genomes (Wu et al., 2019), and it is a simple and powerful gene editing tool (Yang et al., 2021). MicroRNA (miRNA) is a small noncoding single-stranded RNA molecule with a length of approximately 22 nucleotides (Bartel, 2004). Through directly binding to the 3′ untranslated region (UTR) of target gene mRNA, miRNAs induce degradation of mRNA or inhibit mRNA translation, resulting in downregulation of target gene expression. A miRNA can regulate the expression of multiple genes, and a gene can be regulated by multiple miRNAs, making the regulatory network complicated (Tiwari et al., 2018; Chen et al., 2019). It has been shown that aberrant miRNA expression is associated with cancer, including HCC. However, the major network of mRNA and miRNA mediated by HIF1A in HCC remains to be explored.

In order to acquire comprehensive knowledge of *HIF1A-*regulated genes in HCC, in this study, we constructed an *HIF1A* knockout cell model using the CRISPR/Cas9 system. RNA-seq identified differently expressed mRNAs and miRNAs when HIF1A expression was knocked out. We further analyzed the function of HIF1A-regulated mRNA and miRNA.

## Materials and Methods

### Materials

The liver cancer cell lines HepG2 were purchased from the Cancer Institute of the Chinese Academy of Medical Science. HEK293T cells were purchased from ATCC. LentiCRISPRv2 (plasmid ID: #52961, 52961V2 in short.), psPAX2 (plasmid ID: #12260), and pCMV-VSV-G (plasmid ID: #8454) vectors were retrieved from Addgene (Cambridge, MA). The *BsmB*I restriction enzyme was purchased from New England Biolabs (Boston, MA). Genomic extraction kits and total RNA extraction kits were purchased from Omega (Norcross, GA). The reverse transcription kit and qPCR fluorescence MIX were purchased from TAKARA (Dalian, China). The transfection reagent Lipofectamine^®^ 3000 was purchased from Thermo Fisher (Waltham, MA). The antibodies used were HIF-1A (D1S7W, product number: 36169, lot: 2, 1:1000) rabbit monoclonal antibodies (Cell Signaling, United States), beta-actin (13E5, product number: 8457, lot: 7, 1:1000) rabbit monoclonal antibodies (Cell Signaling, United States), and HIF2A (D9E3, product number: 7096, lot: 6, 1:1000) rabbit monoclonal antibodies (Cell Signaling, United States).

### CRISPR/Cas9-HIF1A Plasmid Construction

sgRNAs for *HIF1A* and *LacZ* were designed using CHOPCHOP (http://chopchop.cbu.uib.no). Based on the characteristics of the restriction sites, we added CACCG to the 5′ end and AAAC to the 3′ end (Table 1). After oligo-pairing and annealing, the double-stranded DNA were digested with *Bsmb*I and inserted into the 52961V2 plasmid.

**TABLE 1:** Oligo-sequencing used to construct the CRISPR/Cas9-*HIF1A* and *LacZ* plasmid.

Name		Sequence
*HIF1A*-sgRNA	Forward	5′-CAC​CGA​AGT​GTA​CCC​TAA​CTA​GCC​G-3′
	Reverse	5′-AAA​CCG​GCT​AGT​TAG​GGT​ACA​CTT​C-3′
*LacZ*-sgRNA	Forward	5′-CAC​CGT​GCG​AAT​ACG​CCC​ACG​CGA​TGG​G-3′
	Reverse	5′-AAA​CCC​CAT​CGC​GTG​GGC​GTA​TTC​GCA​C-3′

### Lentivirus Packaging and Transfection

HEK293T cells were transfected with the vector (52961V2--*HIF1A*) and lentiviral packaging vectors (12260; 8454). Supernatants were collected and the lentiviral viruses were concentrated using PEG-it Virus Precipitation Solution (SBI Biosciences).

HepG2 cells were incubated with HEK293 supernatants containing virus plus polybrene at 4 mg/ml at 37°C for 24 h followed by selection with puromycin (for HepG2 cells, 2 μg/ml puromycin).

### Stabilization of HIF1A by CoCl_2_

To stabilize HIF1A protein, HepG2 cells were treated with 0, 50, 100, 150, 200, and 300 μmol/L CoCl_2_ for 24 h. After measurement of cell viability by the MTT kit (Keygen Biotech, China), the expression of HIF1A protein in CoCl_2_-treated cells was examined by Western blot to determine the suitable concentration of CoCl_2_ that can stabilize HIF1A.

### Detection of HIF1A Knockout Efficiency

The target of the wild-type, *LacZ* control, and *HIF1A* knockout HepG2 cells was amplified by PCR and analyzed by Sanger sequencing (Table 2). Knockdown efficiencies were analyzed by comparing treated and control samples with the TIDE webtool (https://tide.nki.nl/). *HIF1A* knockout cell lines with stable expressions were selected. Then, to analyze the protein expression of HIF1A in HepG2 cells transfected with HIF1A gRNA, cells were collected and lysed in RIPA lysis buffer containing proteinase inhibitor, followed by separation by SDS-PAGE, and transferred onto PVDF membranes. After blocking with 5% skim milk in TBST for 2 h, the membrane was incubated with the primary antibody overnight at 4°C followed by washing and incubation with the secondary antibody at room temperature for 60 min. After washing, the membrane was developed with chemiluminescent solution, and the signal was detected by Amersham Imager 600 (General Electric Company, United States). The signal intensity of interest was analyzed using ImageJ software.

**TABLE 2:** Primer list.

Name		Sequence
Primers for *HIF1A* analysis	Forward	5′-TAG​GCC​TTG​TGA​AAA​AGG​GTA​A-3′
	Reverse	5′-GTT​CTG​CAT​TTT​GGA​GAT​CAC​A-3′
*HMGN5* qPCR primer	Forward	5′-CAA​GGT​GAT​ATG​AGG​CAG​GAG-3′
	Reverse	5′-CTT​GAT​GTT​CTT​TTA​GGC​TTC​ACC-3′
*LUM* qPCR primer	Forward	5′-ACC​TTG​AAA​ACT​ATT​ACC​TGG​AGG-3′
	Reverse	5′-GGT​GGA​AGA​CTG​GTT​TCT​GAG-3′
*SLC38A5* qPCR primer	Forward	5′-TTT​TGT​CTG​CCA​CCC​TGA​G-3′
	Reverse	5′-GTA​GAA​GGT​GAG​GTA​TCC​AAA​GG-3′
*MT1E* qPCR primer	Forward	5′-ACT​GCT​TGT​TCG​TCT​CAC​TG-3′
	Reverse	5′-GCT​CTT​CTT​GCA​GGA​GGT​G-3′
*FN1* qPCR primer	Forward	5′-ACT​GTA​CAT​GCT​TCG​GTC​AG-3′
	Reverse	5′-AGT​CTC​TGA​ATC​CTG​GCA​TTG-3′
*IFI6* qPCR primer	Forward	5′-CTG​GTC​TGC​GAT​CCT​GAA​TG-3′
	Reverse	5′-CAC​TAT​CGA​GAT​ACT​TGT​GGG​TG-3′
*OASL* qPCR primer	Forward	5′-GTG​GCA​GAA​GGG​TAC​AGA​TG-3′
	Reverse	5′-CTG​TCA​AGT​GGA​TGT​CTC​GTG-3′
*IL6* qPCR primer	Forward	5′-CCA​CTC​ACC​TCT​TCA​GAA​CG-3′
	Reverse	5′-CAT​CTT​TGG​AAG​GTT​CAG​GTT​G-3′
*JAK3* qPCR primer	Forward	5′-GAC​CTC​AAT​AGC​CTC​ATC​TCT​TC-3′
	Reverse	5′-ATT​CCA​CAG​CCC​ATC​ACG-3′
*PPEF1* qPCR primer	Forward	5′-AGA​AGT​CAT​GGG​ATG​CAG​C-3′
	Reverse	5′-ATG​GTG​AGG​GCA​TAG​TGT​TG-3′
*SERPINE1* qPCR primer	Forward	5′-GTG​GAC​TTT​TCA​GAG​GTG​GAG-3′
	Reverse	5′-GAA​GTA​GAG​GGC​ATT​CAC​CAG-3′
*VEGFA* qPCR primer	Forward	5′-AGT​CCA​ACA​TCA​CCA​TGC​AG-3′
	Reverse	5′-TTC​CCT​TTC​CTC​GAA​CTG​ATT​T-3′
*EFNA3* qPCR primer	Forward	5′-GAA​GTG​TCT​GAG​GAT​GAA​GGT​G-3′
	Reverse	5′-AGT​CTT​CCA​GCA​CGT​TGA​TC-3′
*PGK1* qPCR primer	Forward	5′-GCT​TCT​GGG​AAC​AAG​GTT​AAA​G-3′
	Reverse	5′-CTG​TGG​CAG​ATT​GAC​TCC​TAC-3′
*SLC2A5*qPCR primer	Forward	5′-CCG​TGT​CCA​TGT​TTC​CAT​TTG-3′
	Reverse	5′-ATC​CCA​TTA​AGA​TCG​CAG​GC-3′
*PPFIA4* qPCR primer	Forward	5′-GTA​CCG​CAG​CTA​CTT​CAT​GG-3′
	Reverse	5′-TTC​AGC​CTC​TTC​AGA​CAC​ATG-3′
*LOX* qPCR primer	Forward	5′-ACA​TTC​GCT​ACA​CAG​GAC​ATC-3′
	Reverse	5′-TTC​CCA​CTT​CAG​AAC​ACC​AG-3′
*PFKFB4* qPCR primer	Forward	5′-GTG​CTA​TGA​GAA​CTC​CTA​CGA​G-3′
	Reverse	5′-GAG​GTA​ATA​TAC​GAT​GCG​GCT​C-3′
*SPINK6* qPCR primer	Forward	5′-CAC​AAT​GAA​ACT​GTC​AGG​CAT​G-3′
	Reverse	5′-GTT​AGA​TTC​CCG​AGT​GCA​GTA​G-3′
*UGT1A6* qPCR primer	Forward	5′-TTT​CCT​AAA​GGC​CGG​TCA​TG-3′
	Reverse	5′-TGA​GAC​CAT​TGA​TCC​CAA​AGA​G-3′
β-actin qPCR primer	Forward	5′-TGA​ATG​ATG​AGC​CTT​CGT​GC-3′
	Reverse	5′-CTG​GTC​TCA​AGT​CAG​TGT​AC-3′

### RNA Extraction, cDNA Library Construction, and RNA-Seq

Total RNA was extracted from the *LacZ* control and *HIF1A* knockout HepG2 cells according to the instruction manual of the TRlzol reagent (Life Technologies, California, United States). RNA integrity was assessed using the RNA Nano 6000 Assay Kit of the Agilent Bioanalyzer 2100 system (Agilent Technologies, CA, United States).

The mRNA was isolated from total RNA by the NEBNext Poly(A) mRNA Magnetic Isolation Module (NEB, E7490). The cDNA library was constructed following the manufacturer’s instructions of the NEBNext Ultra RNA Library Prep Kit for Illumina (NEB, E7530) and NEBNext Multiplex Oligos for Illumina (NEB, E7500). In brief, the isolated mRNA was fragmented into approximately 200-nt RNA inserts and used for the synthesis of the first-strand cDNA and the second cDNA. The double-stranded cDNAs were subjected to end-repair/dA-tail and adapter ligation. The suitable fragments were isolated by Agencourt AMPure XP beads (Beckman Coulter, Inc.) and amplified by PCR. Finally, the constructed cDNA libraries of the HepG2 cells were sequenced on a flow cell using an Illumina HiSeq™ sequencing platform.

A small RNA Sample Library Prep Kit for Illumina (NEB, United States) following the manufacturer’s recommendations and index codes was added to attribute sequences to each sample. Briefly, first of all, the 3′ SR adapter was ligated. The 3′ SR adapter for Illumina, RNA, and nuclease-free water is mixed in a mixture system after incubation for 2 min at 70° in a preheated thermal cycle. The tube was transferred to ice. Then, 3′ ligation reaction buffer (2X) and 3′ ligation enzyme mix were added to ligate the 3′ SR adapter and incubated for 1 h at 25°C in a thermal cycler. To prevent adapter-dimer formation, the SR RT primer hybridizes the excess of 3′ SR adapter (that remains free after the 3′ ligation reaction) and transforms the single-stranded DNA adapter into a double-stranded DNA molecule. dsDNAs are not substrates for ligation mediation. Second, the 5′ SR adapter was ligated. Then, reverse transcription synthetic first-chain PCR amplification and size selection were performed. PAGE gel was used for electrophoresis fragment screening purposes, rubber cutting, and recycling as the pieces get small RNA libraries. Last, PCR products were purified (AMPure XP system) and library quality was assessed on the Agilent Bioanalyzer 2100 system. Raw reads in FASTQ format were first processed through in-house Perl scripts.

Finally, clean reads were obtained by removing reads containing adapters, reads containing ploy-N, and low-quality reads from raw data. Then, Q20, Q30, GC-content, and sequence duplication levels of the clean data were calculated. At the same time, miRNAs of reads were trimmed and cleaned by removing the sequences smaller than 18 nt or longer than 30 nt. All the downstream analyses were based on clean data with high quality.

### mRNA-Seq and miRNA-Seq

The clustering of the index-coded samples was performed on a cBot Cluster Generation System using TruSeq PE Cluster Kit v4-cBot-HS (Illumina) according to the manufacturer’s instructions. After cluster generation, the library preparations were sequenced on an Illumina platform and paired-end reads were generated. FPKM data were generated using the fpkm function in DESeq2. Differential expression analysis of two conditions/groups was performed using the DESeq2 R package (1.10.1). DESeq2 provides statistical routines for determining differential expressions in digital miRNA expression data using a model based on the negative binomial distribution. Fold change >1.5 and *p* < 0.05 were considered differentially expressed. The target genes of miRNA were predicted by TargetScan and miRanda.

### Gene Oncology Enrichment Analysis and KEGG Pathway Enrichment Analysis

There are two enrichment analyses used in the current study, Gene Oncology (GO) enrichment and KEGG pathway enrichment. For GO enrichment analysis of the differentially expressed genes (DEGs), the GOseq R package, which is based on Wallenius noncentral hyper-genomic distribution, was used (Young et al., 2010). KEGG is a database resource and can be traced on the website: https://www.genome.jp/kegg/(Kanehisa et al., 2016). Cytoscape software was used for Gene Oncology (GO) enrichment and KEGG pathway enrichment.

### Interaction Analysis of HIF1A Targets

The sequences of the DEGs were blasted to the genome of a related species (the protein–protein interaction of which exists in the STRING database: http://string-db.org/) to predict the interaction of these DEGs which were visualized in Cytoscape.

### Correlation Analysis of mRNA and miRNA

In order to identify potential targets of miRNAs, the R package Hmisc v4.2.0 was used for paired miRNA and mRNA correlation analysis to examine the correlation between miRNA and the predicted target from TargetScan databases. The fsva function in the sva R package was used for frozen surrogate variable analysis to remove nuisance batch effects from both miRNA and mRNA datasets, and the adjusted version of the datasets was used for correlation analysis.

### Real-Time qPCR

Real-time qPCR was run as described. Briefly, *LacZ* control or *HIF1A* knockout HepG2 cells were treated with 100 μmol/L CoCl_2_ for 24 h. Total RNA was extracted using the phenol/chloroform method as described, followed by treatment with DNase I to eliminate residual DNA. cDNA was synthesized using PrimeScript RT Master Mix (Takara Bio) from 2 µg of total RNA according to the manufacturer’s instructions. For miRNA analysis, cDNAs were synthesized using the miRNA First-Strand cDNA Synthesis kit (Sangon Biotech, China) from 2 µg of total RNA. The real-time qPCR using SYBR pre-mix EX Taq (Takara Bio) on qTOWER2.0 (Analytic Jena, Germany) quantified the expression of significantly DEGs according to the manufacturer’s instructions. The thermal cycling procedure started with an initial denaturation at 95°C for 10 min, followed by 45 cycles of denaturation for 10 s at 95°C, primer binding for 20 s at 60°C, and elongation for 20 s at 72°C. The procedure ended with a final amplification at 95°C for 5 s, 65°C for 1 min, the addition of a dissociation curve step, and a cooling step. Primers were purchased from Sangon Biotech (Shanghai, China), and information on primers is shown in Table 2.

### The Cancer Genome Atlas Database Search for Significantly Different Genes

The expression of significantly DEGs in liver cancer and adjacent tissues was analyzed in the TCGA database, and the association between the related DEGs and the level of immune cell infiltration in liver cancer tissues was analyzed.

## Results

### CoCl_2_-Induced Conditions and Plasmid Construct

**FIGURE 1:**
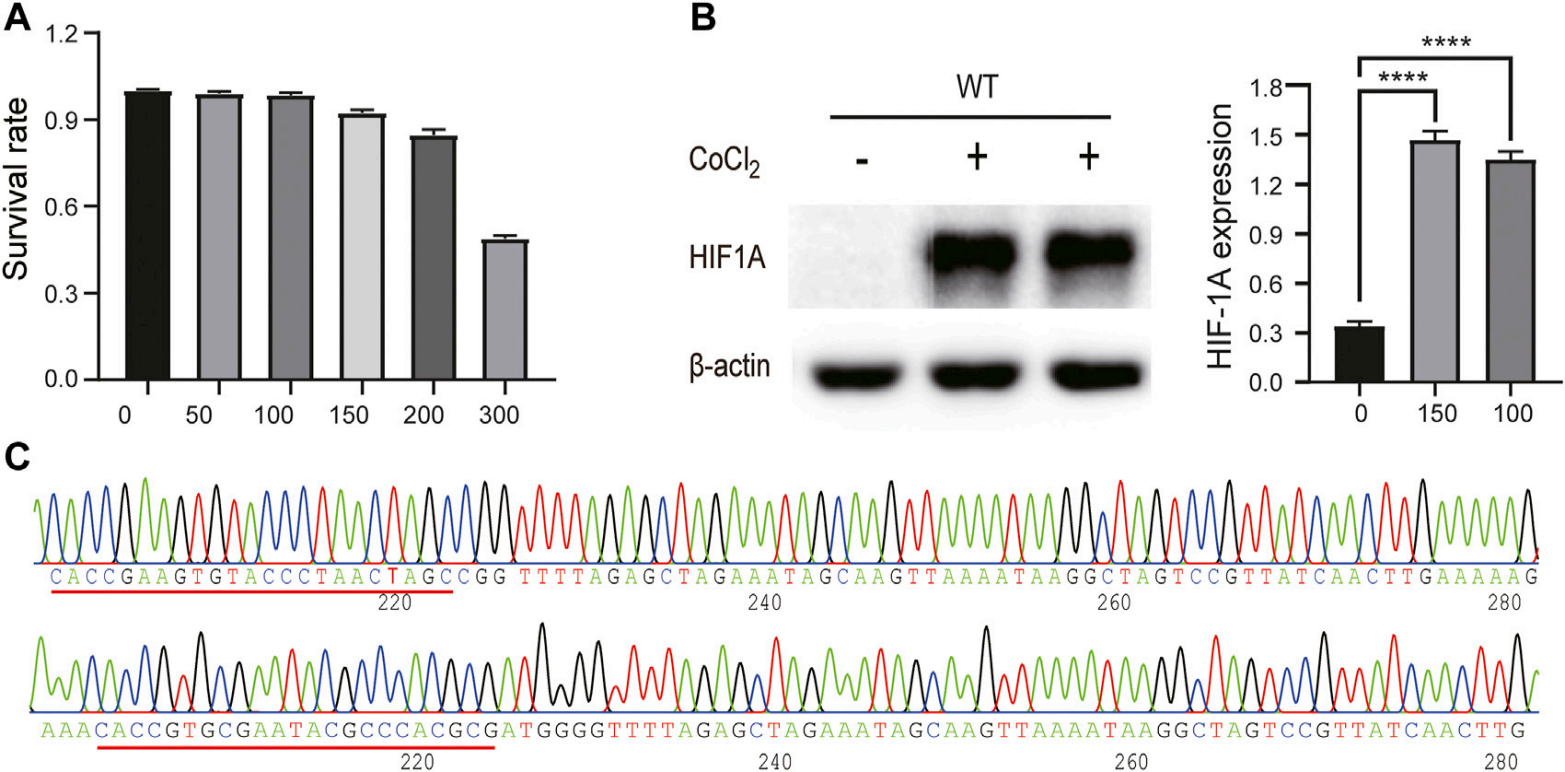
CoCl_2_-induced conditions and plasmid construct. **(A)** Cytotoxicity of CoCl_2_ to HepG2 cells was measured by MTT assay. **(B)** Comparison of HIF1A protein expression between control cells and CoCl_2_-treated cells (****p < 0.0001). **(C)** Sequencing results of 52961V2 plasmid.

In order to enhance HIF1A expression, we treated HepG2 cells with a series of concentrations (0, 50, 100, 150, 200, and 300 μmol/L) for 24 h; in the meantime, we used an MTT assay to monitor the CoCl_2_ toxicity to HepG2 cells. As shown in Figure 1A, cell growth was dramatically inhibited when CoCl_2_ reached 200 μmol/L or higher; we also found that both 100 and 150 μmol/L CoCl_2_ for 24 h could induce HIF1A expression (Figure 1B). Therefore, we selected 100 μmol/L CoCl_2_ to enhance HIF1A expression in the following experiments, and the sequencing results of plasmids 52961-HIF1A and 52961-LacZ showed the same sequences as those of designed sgRNAs, indicating that both plasmids carry the right target sequences (Figure 1C).

### Knockout of HIF1A Expression Using CRISPR/Cas9 in HepG2 Cells

**FIGURE 2:**
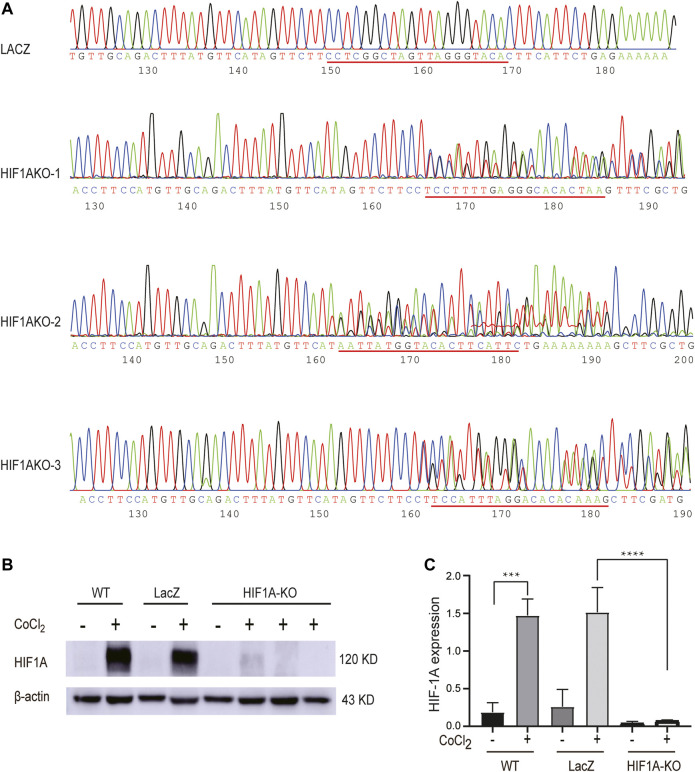
Establishment of *HIF1A*-KO and *LACZ*-control HepG2 cells. **(A)** Stable expression cell lines of 52961V2-HIF1A and 52961V2-LacZ were sequenced. **(B)** Examination of HIF1A protein expression in control cells and HIF1A-KO cells by Western blot. **(C)** HIF1A expression was significantly reduced in the HIF1A knockout group (***p < 0.001, ****p < 0.0001).

We next made an effort on knocking out HIF1A expression in HepG2 cells using the CRISPR/Cas9 technology so as to investigate the downstream targets it potentially impacted in the hypoxic condition. To this end, the sequencing-confirmed vectors were used to produce lentiviruses, which were utilized to infect HepG2 cells. Sanger DNA sequencing results showed that there were indels in those transduced cells resulting from those Cas9 cleavage–induced mutations (Figure 2A), leading to incorrect transcripts of *HIF1A* and subsequent protein depletion, which was confirmed by Western blot (Figure 2B). The results showed that the knockdown efficiency of *HIF1A*KO-3 was the highest, which was used for subsequent experiments.

### Transcriptome Sequencing Reveals There are 1535 mRNAs Differentially Expressed in the HIF1A-Knockout HepG2 Cells

The RNA-sequencing results showed that in the *HIF1A* knockout group, there were 21,490,942, 24,564,111, and 26,423,022 clean reads; in the *LacZ* control group, there were 33,738,934, 39,383,712, and 21,954,987 clean reads. Information on sequencing quality is shown in Table 3. Using the HISAT2 system to compare the clean reads with reference genes (GRCh38/hg38), the *LacZ* control group had a comparison efficiency of 84.65%, and the *HIF1A* knockout had a comparison efficiency of 87.00%.

**TABLE 3:** RNA-seq data statistics.

Samples	Clean reads	Clean bases	GC content (%)	%≥Q20	%≥Q30
*HIF1A*-KO1	21,490,942	6,429,463,828	50.81	98.17	94.68
*HIF1A*-KO2	24,564,111	7,353,232,162	50.39	97.91	95.01
*HIF1A*-KO3	26,423,022	7,904,957,286	50.41	98.10	95.14
*LacZ*-1	33,738,934	10,083,485,654	50.32	98.26	95.19
*LacZ*-2	39,383,712	11,782,502,174	51.97	97.85	94.80
*LacZ*-3	21,954,987	6,561,487,542	50.82	98.19	95.01

**FIGURE 3:**
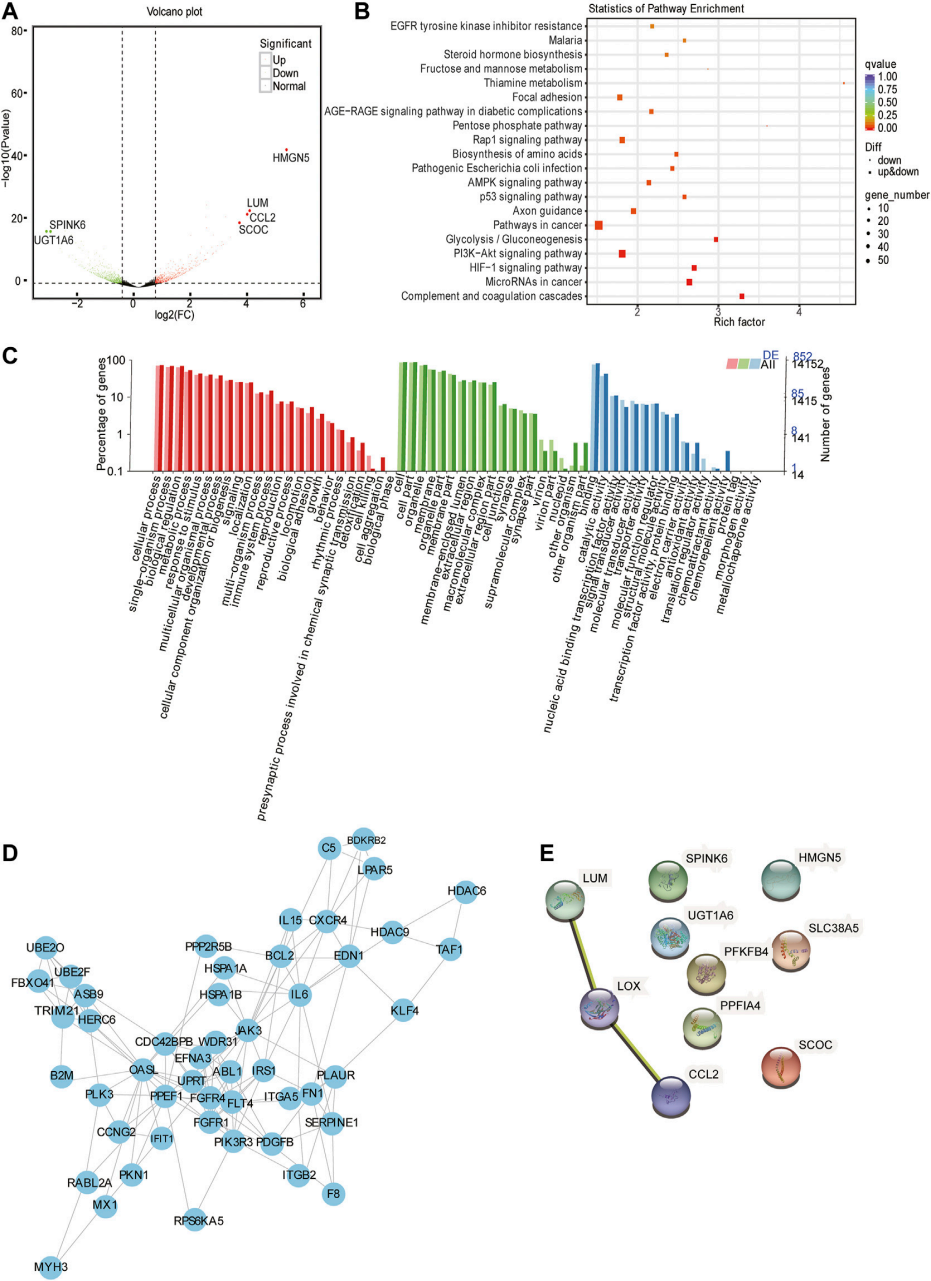
Analysis of mRNA-seq. **(A)** Volcano plot displaying gene expression alterations in HIF1A-KO cells compared to those of the control. The red points represent upregulated DEGs and the blue points represent downregulated DEGs. Black points represent RNAs with no difference in expression. **(B)** Top 20 KEGG pathway enrichment of DEGs. The x-axis indicates the rich factor and the y-axis indicates the pathway names. **(C)** GO analysis of DEGs through diverse GO categories. The red column represents the biological process; the green column represents the cellular component; the blue column represents the molecular function. **(D)** Protein–protein interaction network based on STRING database analysis and Cytoscape. **(E)** PPI analysis of top 10 regulated DEGs.

Gene expression quantification and differential gene analysis showed that in HIF1A knockout cells and control cells, there were 1535 DEGs regulated by HIF1A, of which there were 644 upregulated and 891 downregulated in the HIF1A-knockout HepG2 cells. Figure 3A shows the top six genes with 10-fold differences, and Table 4 listed the top 10 genes whose expressions were found with most differences.

**TABLE 4:** DEGs with most difference in expression when *HIF1A* was knocked out.

Symbol	log_2_FC	*p*-value
HMGN5	6.002	1.27E-80
LUM	5.165	1.27E-43
CCL2	3.865	4.05E-24
SCOC	3.778	5.57E-23
SLC38A5	3.239	8.00E-17
PPFIA4	−2.453	2.33E-10
LOX	−2.589	8.61E-19
PFKFB4	−2.611	1.19E-16
SPINK6	−3.248	1.94E-17
UGT1A6	−3.308	1.67E-17

In order to know the function of HIF1A regulated genes, we next ran GO and KEGG pathway analysis of DEGs. The results showed that there were the top 20 pathways by KEGG metabolic pathway annotation (Figure 3B). In addition, KEGG enrichment uncovered that HIF1A-regulated genes were involved in the “cancer pathway,” “HIF1 signaling pathway,” “PI3K-Akt signaling pathway,” “cancer,” “microRNAs in cancer,” and “P53 signaling pathway.” Furthermore, GO analysis revealed that HIF1A-regulated DEGs were mainly enriched in biological metabolic processes, cell membrane components, catalytic factor activity, transcription factor activity, and other cellular and biological functions (Figure 3C). Notably, *HIF1A* knockout led to a sudden increase in the expression of CCL2 (immune chemokine) and HMGN5 (transcription activator protein) and UGT1A6 and SPINK6 (these genes involved in fat-soluble substance conversion and amino acid degradation). Based on these function assays, we assume that HIF1A can regulate the immune regulation, apoptosis, and inflammatory process of liver cancer cells, and it plays an important role in regulating the energy metabolism of liver cancer cells, such as fatty acids, sterols, and carbohydrates.

To further explore the function of HIF1A-regulated DEGs and their biological roles, we analyzed the DEGs using the STRING database (a database that searches for protein interactions). We obtained a huge network of 587 nodes. K-core is often used as an index in the evaluation of protein correlation. We selected genes from the core node for display (Figure 3D), which contained 50 nodes and a degree of 136. Among these, the five highest connectivity genes were *OASL*, *IL6*, *TAF1*, *JAK3*, and *PPEF1*. We further analyzed 10 DEGs using the STRING database and found genes of *LUM*, *LOX*, and *CCL2* with interaction relationship.

### There are 309 miRNAs Differentially Expressed in the *HIF1A*-Knockout HepG2 Cells

**FIGURE 4:**
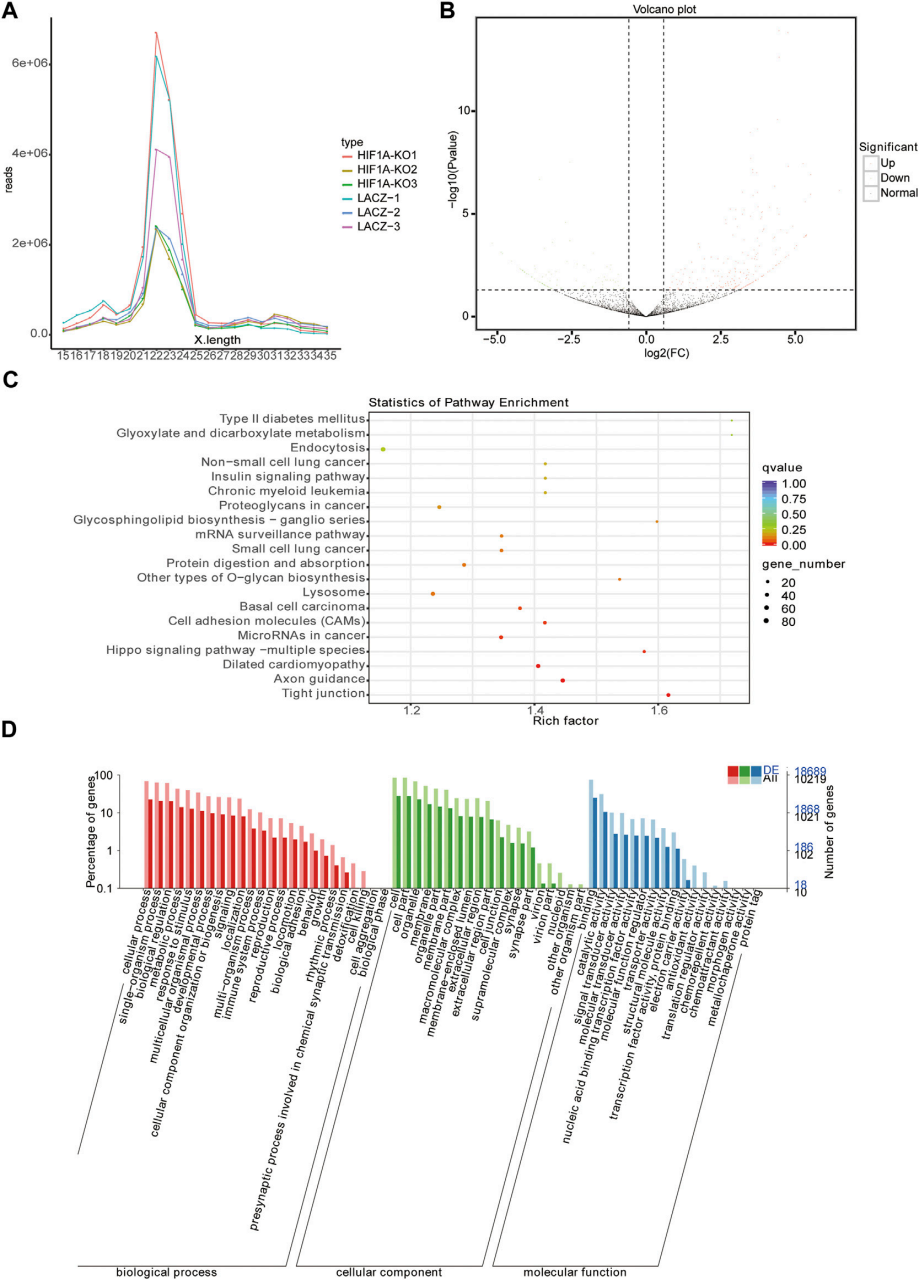
Analysis of miRNA-seq. **(A)** Length distribution of miRNAs in the six libraries. **(B)** Volcano plot displaying miRNA expression alterations in HIF1A-KO cells compared to those of the control. **(C)** Top 20 KEGG pathways enrichment of DEMs. **(D)** GO analysis of DEMs through diverse GO categories.

We also isolated small RNAs from the HIF1A-knockout HepG2 cells and measured their relative abundance using Illumina HiSeq2500 (Biomarker technologies Co, Ltd., Beijing, China). As shown in Table 5, there were clean reads of six samples generated after removing contaminant reads, and it also showed an overview of reads for small RNA sequencing from raw data to high quality and with quality filtering. The lengths of small RNAs were similar among libraries, and there were 21–25 nt RNAs most abundant (Figure 4A).

**TABLE 5:** Summary of sequence data generated of small RNA and quality filtering.

Samples	Raw reads	Length <15	Length >35	Low quality	Containing “N” reads	Clean reads	Q30 (%)
HIF1A-KO1	10,459,583	249,673	647,206	0	0	9,562,704	96.88
HIF1A-KO2	12,646,277	294,261	639,410	0	0	11,712,606	96.18
HIF1A-KO3	12,375,420	216,731	704,546	0	0	11,454,143	97.23
LacZ-1	15,985,038	157,581	902,072	0	0	14,925,385	96.75
LacZ-2	14,496,411	222,378	659,882	0	0	13,614,151	97.33
LacZ-3	17,798,204	254,938	751,238	0	0	16,792,028	97.62

Using miRNA sequencing, we identified 65 known miRNAs and 244 novel miRNAs in total as HIF1A-regulated miRNAs in the HIF1A-knockout HepG2 cells (Figure 4B). Of these 309 DEMs (differentially expressed miRNAs), the expressions of 213 miRNAs were upregulated (expression of 100 miRNAs increased more than 10-fold) and those of 96 miRNA decreased (expression of 38 miRNAs decreased more than 10-fold) in the HIF1A-knockout HepG2 cells. Notably, the top 12 DEMs (including six known miRNAs and six novel miRNAs) are listed in Table 6.

**TABLE 6:** DEMs whose expressions are regulated when HIF1A expression was knocked out.

Symbol	log_2_FC	*p*-value
novel_miR_2837	6.491	7.24E-07
novel_miR_984	5.531	1.23E-07
novel_miR_3081	5.529	1.10E-06
hsa-miR-1248	3.881	1.08E-02
hsa-miR-522-3p	3.727	1.68E-03
hsa-miR-145-3p	3.680	1.69E-02
novel_miR_3118	−4.818	8.42E-04
novel_miR_2903	−4.864	7.31E-04
novel_miR_1569	−5.163	2.67E-04
hsa-miR-514a-3p	−3.464	2.49E-02
hsa-miR-5191	−4.194	5.06E-03
hsa-miR-1298-5p	−4.518	1.96E-03

To elucidate the biological functions of the identified DEMs, we used miRmap and TargetScan to predict their target genes and found there were 11,889 genes as the potential miRNA targets. KEGG pathway analysis and GO function annotations of the 11,889 genes showed that they were enriched in the ferroptosis pathway. KEGG enrichment analysis discovered the first five pathways as “tight junction,” “axon guidance,” “dilated cardiomyopathy,” “hippo signaling pathway-multiple species,” and “microRNAs in cancer” (Figure 4C). GO analysis further showed that they were involved in the single-organism process, cell part, and catalytic activity (Figure 4D).

**FIGURE 5:**
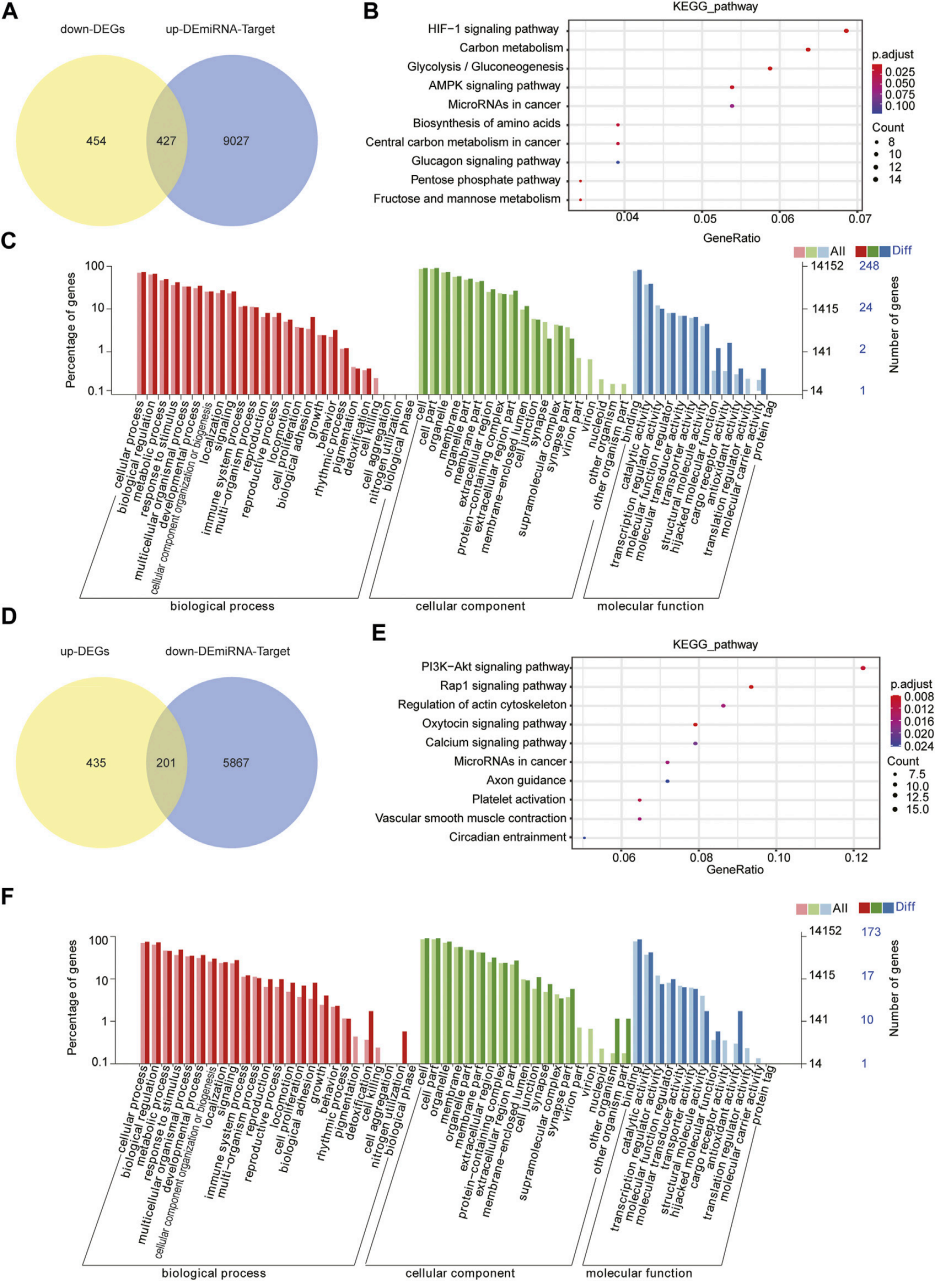
Joint analysis of miRNA and mRNA. **(A)** Intersection of target genes of upregulated DEMs and downregulated DEGs. **(B)** Top 10 KEGG pathways the target genes are enriched in. **(C)** GO analysis of target genes. **(D)** Intersection of target genes of downregulated DEMs and upregulated DEGs. **(E)** Top 10 KEGG pathways enrichment of D **(F)** GO analysis of D through diverse GO categories.

To clarify the regulatory relationship between miRNAs and mRNAs, we identified the potential miRNA–mRNA pairs based on gene expression profiles obtained earlier. Usually, miRNAs negatively regulate the expression of their target genes, that is, increased miRNA expression leads to downregulation of target genes and vice versa. As shown in Figure 5A, we identified 425 genes by the intersection of downregulated DEGs and target genes of upregulated DEMs. KEGG pathway analysis and GO function annotations of these genes revealed that they were enriched in the HIF-1 signaling pathway, carbon metabolism, and glycolysis/gluconeogenesis (Figure 5B). In GO analysis, these genes were mainly enriched in biological regulation, cell part, and catalytic activity (Figure 5C). Furthermore, through the intersection of upregulated DEGs and target genes of downregulated DEMs, we identified 201 genes (Figure 5D), of which KEGG enrichment analysis showed their enrichment in the first three pathways, “PI3K-Akt signaling pathway,” “Rap1 signaling pathway,” and “Oxytocin signaling pathway” (Figure 5E), and GO analysis showed their enrichment in the single-organism process, cell part, and catalytic activity (Figure 5F).

### qPCR Assays and the Analysis of The Cancer Genome Atlas Database

**FIGURE 6:**
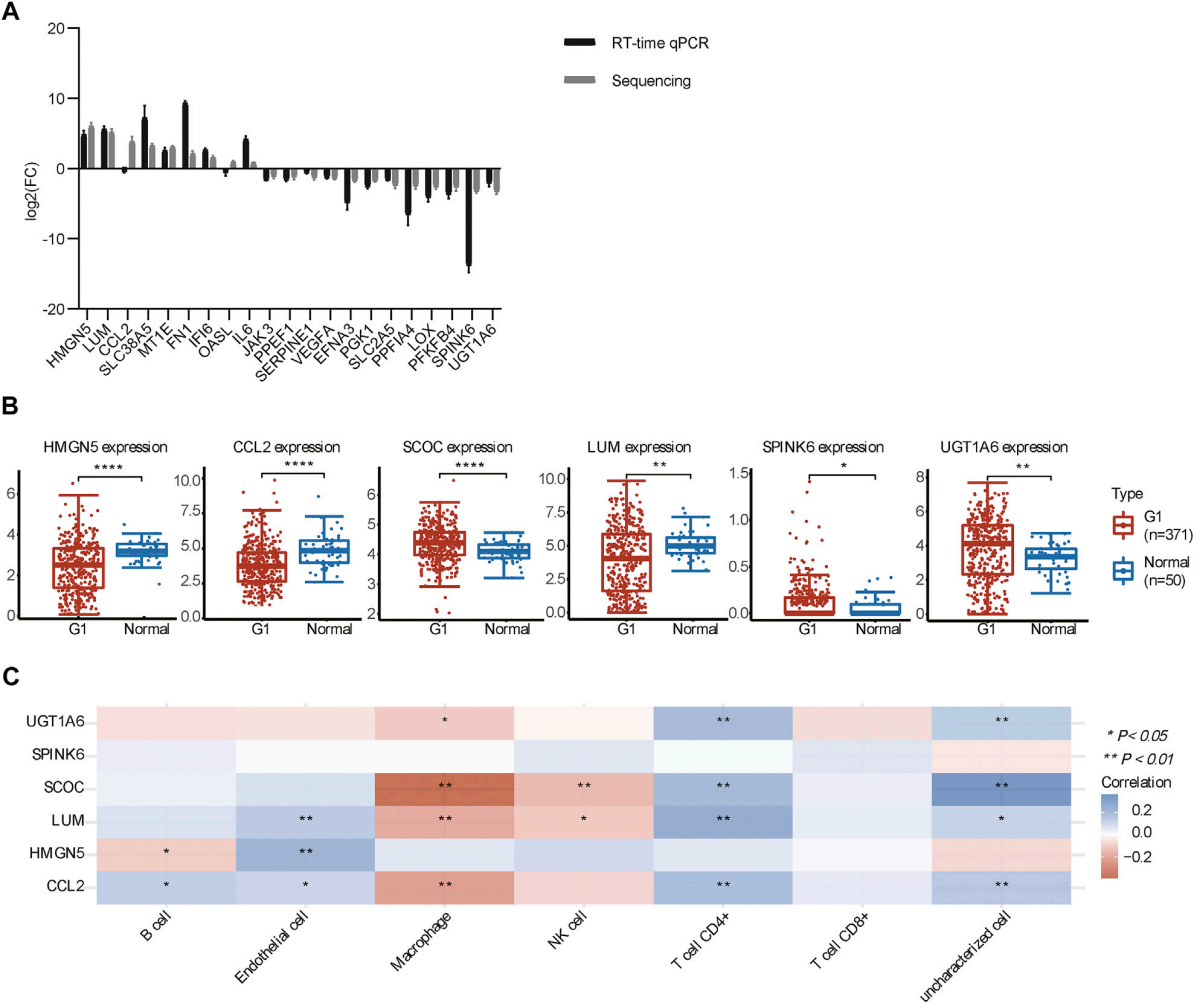
DEG expression analysis by RT-qPCR and in TCGA. **(A)** Expression of DEGs in the *HIF1A*-KO and *LACZ*-control HepG2 cells. **(B)** Expression of DEGs in tumor and normal tissues in TCGA (*p < 0.05, **p < 0.01, and ****p < 0.0001). **(C)** Heat map of the correlation between multiple genes and immune cells in HCC. The horizontal and vertical coordinates represent genes, different colors represent correlation coefficients (blue represents positive correlation and red represents negative correlation), and the darker color represents the two stronger correlations (*p < 0.05 and **p < 0.01).

To verify the RNA-seq results, we examined 21 representative genes by qPCR, and the results showed that the overall trend of qPCR and RNA-seq follows approximately the same pattern (Figure 6A), indicating that the sequencing results were credible. We further examined the DEGs (fold change >10) in 50 adjacent normal tissues and 371 HCC samples from TCGA. The analysis revealed that the expression of *UGT1A6*, *SPINK6*, and *SCOC* was stronger in HCC than in adjacent normal tissues, while the expression of *HMGN5*, *LUM*, and *CCL2* was weaker in HCC than in adjacent normal tissues (Figure 6B). To this end, we used the R software package to display the multi-gene correlation map and discovered that *LUM*, *SCOC*, and *CCL2* were significantly associated with immune cell infiltration levels.

## Discussion

HCC is the fifth most common malignancy with high mortality worldwide, accounting for approximately 90% of primary liver cancers, and the five-year survival rate is less than 10% (Anwanwan et al., 2020). It is known that hypoxia is a common characteristic of solid tumors including HCC, and hypoxia is essential for cancer initiation, progression, metastasis, and drug resistance (Gonzalez et al., 2018; Mendez-Blanco et al., 2018; Feng et al., 2020). HIF1A is a key regulator of the hypoxia responses (Song et al., 2018), regulating the expression of target genes and miRNAs that play an important role in angiogenesis, erythropoiesis, energy metabolism, and cell survival (Ampuja et al., 2017). In particular, ADM, VEGF, P53, miR-18a, miR-199a, and miRNA-376b-5p are regulated by HIF1A (Loboda et al., 2010; Dai et al., 2015; Serocki et al., 2018; Yang et al., 2019).

The regulation of HIF1A is a complex network. So far, a comprehensive miRNA–mRNA regulatory network of HIF1A in HCC has not been reported yet. In this present study, we established a *HIF1A* knockout and *LacZ* control model using the CRISPR/Cas9 technology in HepG2 cells. Using RNA-seq, we screened the mRNAs and miRNAs with differential expressions. Then, 1535 DE-mRNAs and 309 DE-miRNAs (65 known miRNAs and 244 novel miRNAs) were identified. According to the gene function annotations, HIF1A-related pathways were identified. In particular, the cancer pathway, microRNAs in cancer, PI3K-Akt signaling pathway, and HIF1A signaling pathway were enriched in the analysis of transcriptome sequencing. In addition, the hippo signaling pathway, axon guidance, and tight junction were enriched in miRNA transcript levels. In the integration analysis of miRNA–mRNA expression profiles, we constructed a miRNA–mRNA regulatory network according to the DEM and DEG datasets and miRNA-targeting information. We found that the HIF-1 signaling pathway, carbon metabolism, and glycolysis/gluconeogenesis were enriched in the integration analysis of downregulated DEGs and target genes of upregulated DEMs and the PI3K-Akt signaling pathway,” “Rap1 signaling pathway,” and “oxytocin signaling pathway” were enriched in the integration analysis of upregulated DEGs and target genes of downregulated DE-miRNAs. These data confirm the important role of HIF1A in tumor cell proliferation and apoptosis.

Our further analysis uncovered that both UGT1A6 involved in lipolysis SPINK6 that involved in amino acid degradation were decreased significantly (Kobayashi et al., 2006; Ge et al., 2017), suggesting that HIF1A is involved not only in the glycolytic pathway (Hu et al., 2006) but also in the other energy metabolism processes. Furthermore, the chemokine CCL2 and nucleosome-binding protein HMGN5 were increased significantly since CCL2 plays a key role in tumor inflammation and HMGN5 might be the important regulators of gene expression in cells (Tang et al., 2012; Lin et al., 2013; Gonzalez-Romero et al., 2015; Liu et al., 2020). These data suggest that immune responses in cancer are also regulated by HIF1A. It is intriguing that the expressions of HMGN5 and CCL2 are greatly influenced by HIF1A, but how these two genes are regulated by HIF1A still remains to be clarified.

**FIGURE 7:**
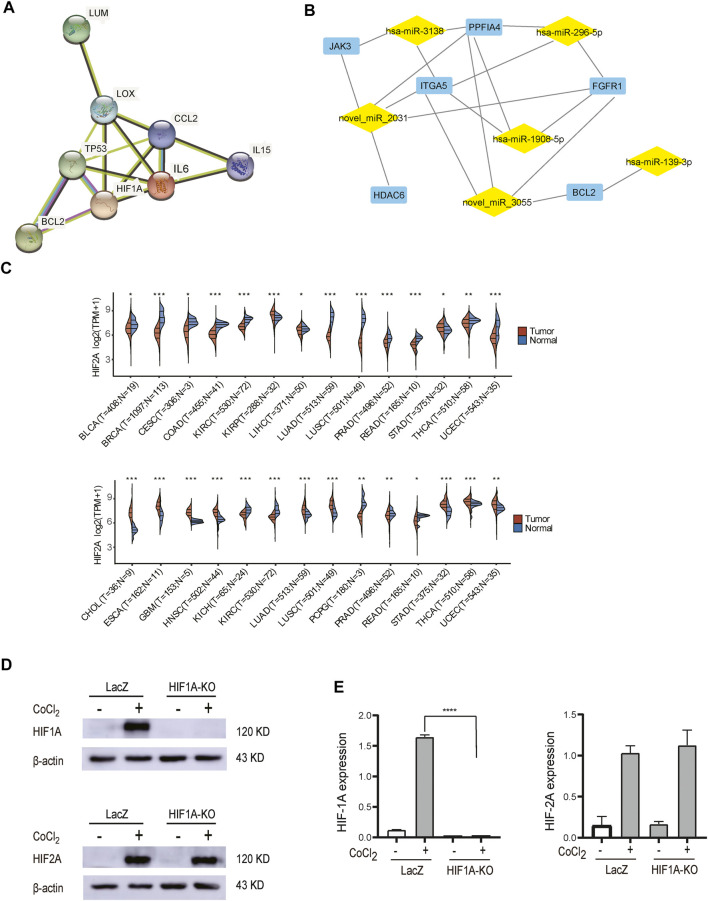
Potential mechanistic. **(A)** PPI of hub genes. **(B)** Putative miRNA–mRNA correlation network. Expression of HIF1A and HIF2A in pan-carcinomatissues (*p < 0.05, **p < 0.01, ***p < 0.001). **(D)** Examination of HIF2A protein expression in control cells and HIF1A-KO cells by Western blot. **(E)** The HIF2A expression of HIF1A-KO and LACZ-control HepG2 cells was not changed significantly (****p < 0.0001).

The identified PPI network and top 50 hub genes included angiogenesis-related genes, such as FGFR1, FGFR4, FDGFB, and EDN1 ; immune-related genes such as IL6, JAK3, and IL15 ; and some associated with apoptosis and drug resistance such as ABL1, BCL2, PLK3, and PAF1. Among the 10 most regulated DEGs analyzed by PPI analysis, these three genes, *LUM*, *LOX*, and *CCL2*, have interaction effects. After comprehensive analysis, we established a staggered network (Figure 7A), suggesting that HIF1A has great association with the pathway, and this pathway might prove to be useful in the diagnosis, treatment, and prognosis of HCC patients. In addition, by integrated analysis of mRNA and miRNA, we have also established a potential network, which shows some networks of node genes (Figure 7B). In this network, PFKFB4, HMGN5, FGFR1, BCL2, JAK3, LOX, ITGA5, miR-296, and miR-145 have been demonstrated to associate with HIF1A (Minchenko et al., 2004; Preusser et al., 2014; Sun et al., 2018; Zhao et al., 2018; Gentile et al., 2019; Xu et al., 2019; Saatci et al., 2020; Li and Li, 2022), these molecules are closely related to inflammation, tumor metastasis, apoptosis, and drug resistance; however, the mechanism of action whereby these regulatory factors act on the HCC remains to be elucidated. In conclusion, we have discovered many more putative HIF1A target mimics, important for exploring novel mechanisms and therapeutic targets.

Some studies show that HIF2A was also particularly important under hypoxia, and there is expression crosstalk between HIF2A and HIF1A (Branco-Price et al., 2012; Kocabas et al., 2012; Krzywinska et al., 2017; Hsu et al., 2020). We performed a pan-cancer search for HIF1A and HIF2A through TCGA databases, and HIF1A and HIF2A were found to be differentially expressed in many same or different cancers (Figure 7C). The expression of HIF2A was confirmed in HIF1A-KO and *LACZ*-control HepG2 cells by WB analysis. The results showed that HIF2A was not changed significantly (Figure 7E). This suggests that the functions of HIF1A and HIF2A may be relatively independent in HepG2 cells. Therefore, in the follow-up study, we plan to obtain HIF2A knockout cell lines for RNA-seq sequencing and compare them with this experimental result.

Finally, there are some limitations in this study, namely, the results we obtained need to be verified by other experimental methods, and novel miRNAs identified need to be further explored to understand the underlying molecular mechanism. In summary, the present study delineates a network that may help explore the molecular mechanisms of HIF1A in HCC, providing clues for the novel therapeutic targets for HCC treatment.

## Data Availability

The original contributions presented in the study are publicly available. This data can be found here: PRJNA79920.
